# Time trends in cardiovascular disease mortality in Russia and Germany from 1980 to 2007 - are there migration effects?

**DOI:** 10.1186/1471-2458-10-488

**Published:** 2010-08-17

**Authors:** Andreas Deckert, Volker Winkler, Ari Paltiel, Oliver Razum, Heiko Becher

**Affiliations:** 1 Institute of Public Health, University of Heidelberg, INF 324, 69120 Heidelberg, Germany; 2 Central Bureau of Statistics, Jerusalem, Israel; 3 Faculty of Health Sciences, Bielefeld School of Public Health, Bielefeld, Germany

## Abstract

**Background:**

Cardiovascular disease (CVD) is the leading cause of death in the industrialized world. Large variations in CVD mortality between countries and also between population subgroups within countries have been observed. Previous studies showed significantly lower risks in German repatriates and Jews emigrating from Russia than in the general Russian population. We examined to what degree the migration of large subgroups influenced national CVD mortality rates.

**Methods:**

We used WHO data to map the CVD mortality distribution in Europe in 2005. Supplemented by data of the Statistisches Bundesamt, the mortality trends in three major CVD groups between 1980 and 2007 in Russia and Germany are displayed, as well as demographic information. The effects of migration on demography were estimated and percentage changes in CVD mortality trends were calculated under the assumption that migration had not occurred.

**Results:**

Cardiovascular disease mortality patterns within Europe showed a strong west-east gradient with ratios up to sixfold. In Germany, the CVD mortality levels were low and steadily decreasing, whereas in Russia they fluctuated at high levels with substantial differences between the sexes and strong correlations with political changes and health campaigns. The trends in both Russia and Germany were affected by the migration that occurred in both countries over recent decades. However, our restricted focus in only adjusting for the migration of German repatriates and Jews had moderate effects on the national CVD mortality statistics in Germany (+1.0%) and Russia (-0.6%).

**Conclusions:**

The effects on CVD mortality rates due to migration in Germany and Russia were smaller than those due to secular economical changes. However, migration should still be considered as a factor influencing national mortality trends.

## Background

Cardiovascular disease (CVD) is the most frequent cause of death in the industrialized world [1] and is often a consequence of an arteriosclerotic process with a complex background. The main risk factors for CVD are hypercholesterolemia, smoking, diabetes mellitus and hypertension [2,3]. Aside from these, a contribution by genetic factors has been demonstrated [4-6]. Besides life-style related factors, socioeconomic status also plays a role [7,8]. Thus, the major CVD risk factors in developed nations have been identified and suitable prevention strategies as well as treatment procedures are implemented in these populations.

Cardiovascular disease mortality has a distinct spatial and temporal pattern. The variation within Europe is very broad. While there has been a strong decline in many western countries over the last few decades, in other parts of Europe the pattern is not as pronounced, and in Eastern Europe increases in CVD mortality have been observed [9,10].

Cardiovascular disease morbidity and mortality varies both between and within countries, and wide variations between ethnic and socioeconomic subgroups have even been observed within countries. In the Former Soviet Union (FSU), for instance, a variety of distinct population subgroups existed with pronounced visible differences in their mortality patterns [11,12].

The prevalence of risk factors such as alcohol consumption and smoking is generally higher in former socialist countries in the eastern part of Europe than in the west [13-15]. In Russian men aged 25-34, the smoking prevalence in the late 1990s was about 73% [14]. Worldwide, the highest proportion of heavy drinkers (18.9%) is in the region of Russia and the Ukraine, which is combined with conspicuous patterns of drinking behaviours [15-17]. When considering life-style related risk factors like nutrition, people in Russia are known to consume high amounts of red meat and saturated fat combined with a low intake of fruit and vegetables [18,19].

In addition to these risk factors, physical and mental stress caused by migration could also affect the development of CVD [20]. Giving up a familiar social environment is known to play a role in the emergence of psychological and psychiatric conditions [21]. Long-term exposure to continuous stress may increase the risk of CVD mortality [22].

In- and out-migration in Russia and Germany:

The legal successor state of the Soviet Union is the Russian Federation, in which ethnic Russians comprise about 80% of the population. To simplify matters we will refer to it as Russia. After a steady and long-term population growth, peaking at 148.8 million in 1992, the population of Russia experienced rapid decline from the mid-1990s, mainly due to a steadily increasing number of deaths combined with a rapidly decreasing number of births in the late 1980s and early 1990s (see chapter results) [23]. Concomitantly, immigration to Russia by far exceeded emigration after the collapse of the FSU (especially between 1991 and 1995), which attenuated the negative balance of births and deaths [24]. Between 1992 and 1998 net migration was about 3.6 million [25].

In Germany, rates of emigration since 1980 only exceeded those of immigration between 1981 and 1984. In later years, rates of immigration increased considerably with net migration reaching a peak in 1992, followed by a decrease in immigration with nearly stable but comparatively lower emigration rates. The large number of immigrants was mainly composed of groups of ethnic German repatriates [26]. Altogether, about 4.5 million German repatriates migrated to Germany between 1950 and 2007. Between 1991 and 2007, German repatriates accounted for nearly 50% of all migrants. Between 1989 and 2007, approximately 2.19 million of all German repatriates came from the FSU, of whom about 824,000 were from Russia (based on estimates for the years before 1992) [27].

Previous studies of German repatriates and Jewish FSU migrants reported significantly lower overall CVD mortality rates compared both to the German and Israeli populations, and to that of Russia as well [28,29]. The lower CVD risk cannot be explained by known risk patterns. In the case of German repatriates these findings cannot be explained by a healthy migrant effect, since almost the whole population of ethnic Germans in Russia resettled in Germany rather than just a selection of relatively healthy individuals.

In this paper, we examined the most recent CVD-related mortality patterns in Europe (2005) and time trends with an emphasis on Germany and Russia. Since both countries experienced considerable in- and out-migration of subgroups whose CVD mortality was apparently different to that of their majority populations, we estimated the effect of migration on the overall mortality pattern.

## Methods

### Data sources

We used time series of mortality data provided by the World Health Organisation's (WHO) European Health for All Database (HFA-DB) and the Statistisches Bundesamt (SB). Data from the WHO for Germany is only available between 1990 and 2006. As the WHO data are based on data from the SB, we used data from the SB for Germany to cover a wider time period. Rates were standardized using the Old European Standard Population. The causes of death in these databases are coded in ICD10.

The population size of Russia was obtained from the WHO database. German population figures were based on SB data. Population data of German repatriates was obtained from the Bundesverwaltungsamt (BVA) in Cologne. Data on German repatriates from the FSU, Poland and Romania are available from 1950 onwards. Data relating to Russia are available from 1992 onwards. We used data from the Central Bureau of Statistics in Israel to obtain the population size of the Jewish FSU migrants to Israel, which was available for the years 1990 to 2006.

Interpreting time trends and discussing possible influences affecting these time trends requires consideration of other demographic factors, for example birth and death rates. This additional information was also gathered from the HFA-DB.

**Figure 1:**
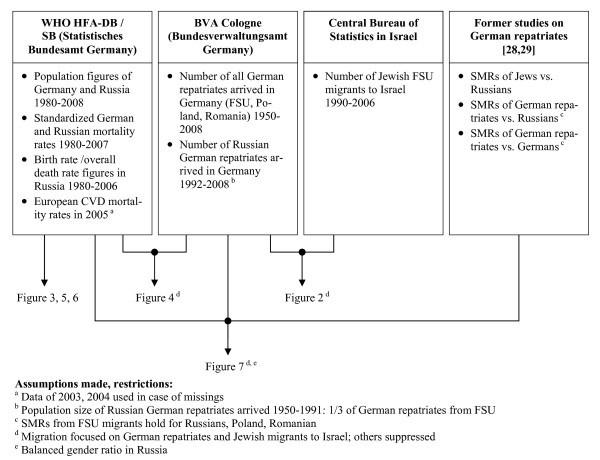
**Data sources and data flow**.

All the data used are publically available. Figure 1 illustrates the data sources and data flow.

### Study population

**Figure 2:**
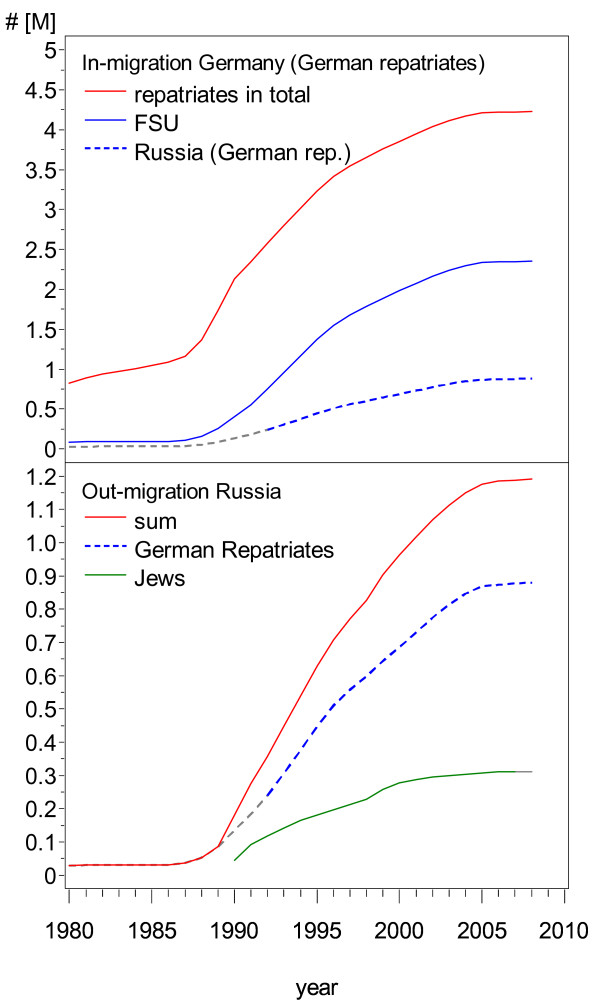
**In-migration into Germany and out-migration from Russia of study-relevant population subgroups (cumulative numbers)**. German repatriates from Russia: data between 1980 and 1991 uncertain, estimated to be one third of the total number from the FSU.

We summed up the annual number of repatriates who arrived in Germany. The total arrivals of German repatriates from the FSU into Germany add up to 2,352,044 until 2008. Considering Poland and Romania, 4,227,266 German repatriates moved in. Detailed data on the repatriates from Russia are available since 1992. We assumed that migrants originating from Russia constituted one third of all repatriates from the FSU before 1992. No data are available to support this assumption between 1980 and 1989; however, the total numbers are low and the effect is negligible. Thus, a total of 879,550 immigrants arrived from Russia by 2008 (see Figure 2).

Overall, 311,087 individuals migrated from Russia to Israel between 1990 and 2006, with the numbers rapidly decreasing over that time. Data before 1990 are not available but the numbers are likely to be small (see Figure 2).

### Statistical analyses

To illustrate the CVD mortality pattern in Europe in 2005, age-adjusted mortality rates were mapped (by sex). Time trends of total population sizes and age-adjusted mortality rates (total CVD, ischaemic heart diseases and cerebrovascular diseases) in Russia and Germany are presented by sex, between 1980 and 2007.

We calculated an if-then-scenario to estimate the size of the migration effect. First, we adjusted the total population size in Russia for each year, including all Jews and the German repatriates who left Russia and its parent part of the FSU. We also adjusted the German population for each year by subtracting German repatriates, including all German repatriates from Russia, Poland and Romania. Furthermore, we assumed that all German repatriates had the same mortality rates as the repatriates from the FSU as estimated from previously mentioned migrant studies [28,29]. In a second step, we recalculated the expected mortality if German and Jewish migrants had stayed in Russia. To do so, we multiplied national mortality rates with the particular SMR estimate. In the scenario for Russia, the SMRs of the subgroups in relation to the population of Russia were used, and in the scenario for Germany the SMRs in relation to the general German population were used. Specifically, the reported SMR for the overall CVD of Jewish FSU migrants compared to the Russian population was 0.263 (95% CI: 0.258-0.269) in males and 0.30 (0.29-0.31) in females [30,31]. The SMR of German FSU migrants compared to the German population was 0.76 (0.69-0.84) and 0.84 (0.77-0.91) in males and females, respectively, and compared to the Russian population an SMR of 0.27 (0.25-0.30) in males and 0.38 (0.35-0.41) in females was reported. We assumed that the mortality risk ratios were constant over the calendar period 1980-2007. We then calculated corrected national mortality rates assuming no migration for both Germany and Russia, weighting the known national mortality rates and the calculated rates for the migrant subgroups according to the ratio of the specific population sizes in the total population. In a final step, we calculated the percentage changes that would have been seen in the mortality trends if migration had not taken place.

## Results

**Figure 3:**
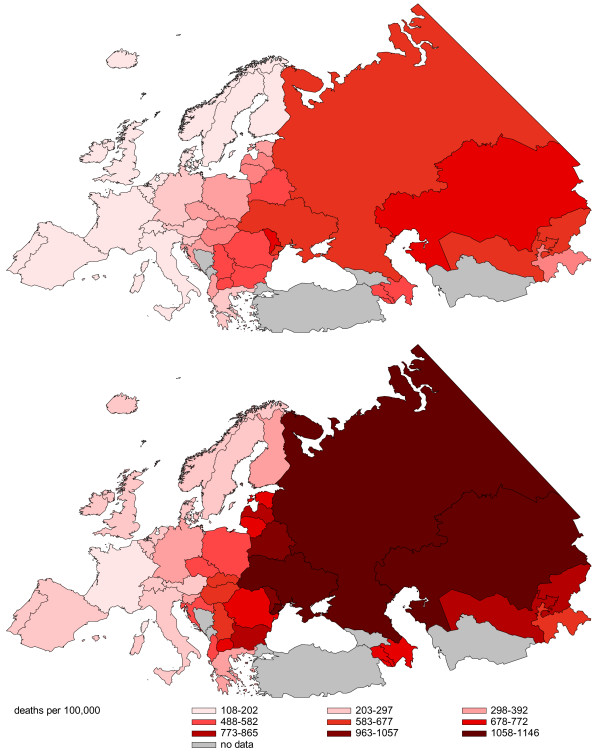
**Age-standardized CVD mortality in Europe in 2005 (ICD-10: I00-I90)**. Top: females, bottom: males. Values of 2003 imputed: Armenia, Macedonia, Italy; values of 2004 imputed: Albania, Belgium, Azerbaijan, Portugal.

Figure 3 shows the CVD mortality pattern in Europe in 2005 (and neighbouring years for some countries). The CVD mortality rates rise steeply from west to east with the largest values in Russia and its bordering countries. The lowest rates are found in France (females 109.88, males 186.17), the highest rates for males in Russia (1145.11), and the highest rates for females in the Republic of Moldova (750.2; see Figure 3). The ratio between the highest and lowest rates is about 6:1.

**Figure 4:**
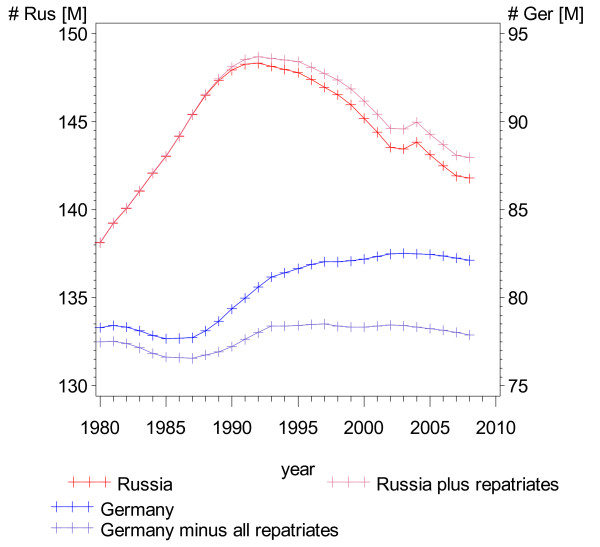
**Demographic trends: scenario-corrected population figures, Russia and Germany**.

Figure 4 shows the population figures of Russia and Germany both as observed and under the assumption of the no migration scenario. The trend in the German population size was greatly influenced by the large group of German repatriates (Figure 4).

**Figure 5:**
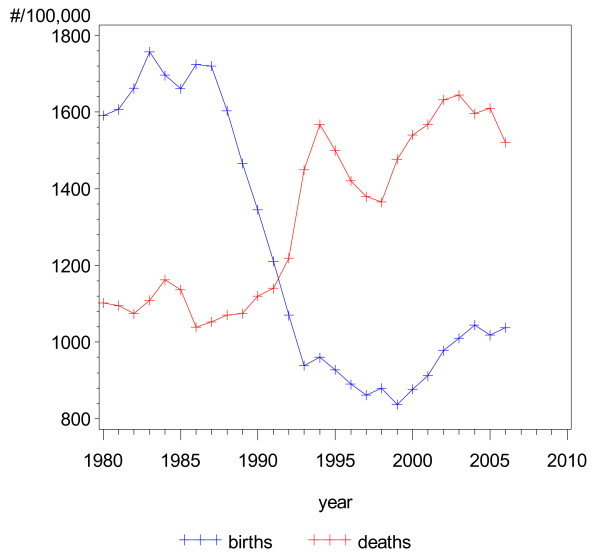
**Demographic trends: birth and death rates, Russia**.

The Russian population increased by about 10 million between 1980 and 1990, whereas after the collapse of the Soviet Union the population decreased by approximately 6 million people. This cannot be explained by the emigration of German repatriates and the Jewish migrants, or by emigration to other neighbouring countries, as the net migration was positive. The cause of the decline was a massive drop in the birth rate combined with a strong increase in overall mortality in the years just before and after the breakup of the Soviet Union in 1991 (Figure 5).

**Figure 6:**
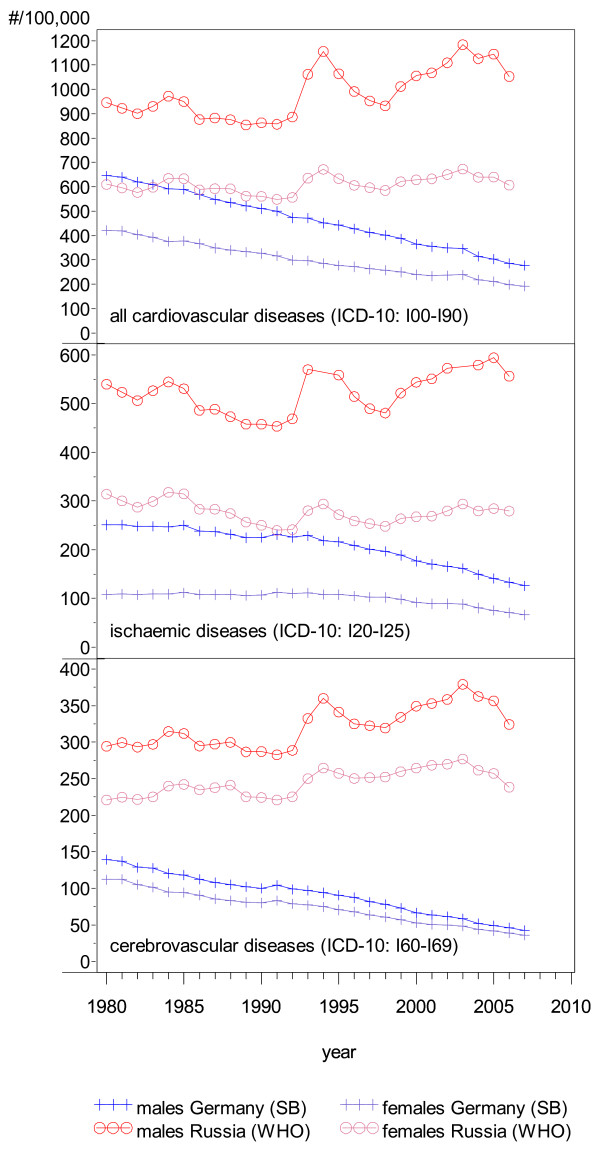
**Age-standardized trends in mortality from cardiovascular diseases and subgroups, Russia and Germany, by sex**. (WHO: Old European Standard Population; SB: Old European Standard Population).

The distinct jag in the mortality rate trend with a steep increase between 1991 and 1994, followed by a partial recovery and then another increase in recent years can be seen in all of the CVD-related mortality trends (Figure 6).

In Germany there was a 50% reduction in overall CVD-related mortality from 1980 to 2007, with a constant trend over time and decreasing disparities between males and females. In contrast, the pattern in Russia is less clear with stable and large differences between the sexes.

Considering ischaemic diseases separately, a peculiarity can be seen in Germany (Figure 6): after 1990 the downwards trend seems to be steeper for males than for females. In Russia, the patterns for ischaemic diseases, cerebrovascular diseases and overall CVD-related mortality are similar (Figure 6).

**Figure 7:**
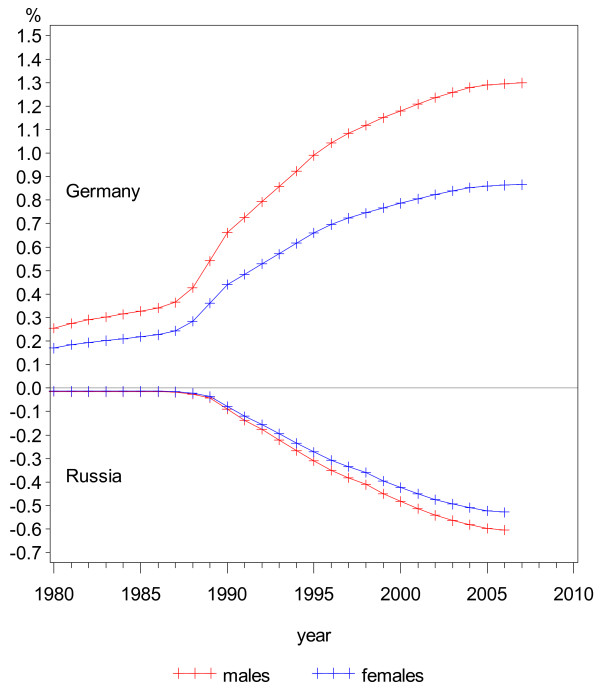
**Estimated percentage changes in CVD mortality rates due to migration, Russia and Germany, by sex**.

Correcting the CVD mortality rates in Russia and Germany assuming no migration did not have a large impact, as indicated by the percentage changes in Figure 7. The effect, however, increases continuously over time, since population figures are accumulating, while the yearly change is largest around 1990 when migration volumes were highest. There were larger differences in CVD mortality rates between the sexes in Germany, which was caused by the wider differences in the reported SMRs and by the higher percentage change in the population. The rate of CVD mortality in Germany in 2006 would have been about 1% higher if no immigration of repatriates had occurred. In Russia, despite the low CVD rates of the migrating population, the overall rates would have been only 0.6% lower if no migration had occurred. For both the German repatriates and the Jews, the SMR estimates for males are lower than for females, and therefore the percentage change in male mortality is higher in both directions (since the subgroups were added in Russia and subtracted in Germany).

## Discussion

We showed that in- and out-migration may have an effect on the mortality trends of countries with large migration movements. We could find only small effects because we only focussed on two of many relevant migrant groups and did not consider the complexity of migration processes, but our findings do show that migration is an additional factor influencing mortality trends. This should be taken into consideration when interpreting mortality trends in nations with large immigration and emigration movements. Furthermore, we presented the most recent picture of the west-east gradient in overall CVD mortality rates. Additionally, we showed the trends in CVD mortality rates in three major CVD disease groups between 1980 and 2007, with an emphasis on Germany and Russia as representatives of West and East Europe, demonstrating the huge differences in time trends which have brought about the current differences.

Given the large differences in CVD mortality between the overall population of Russia and the specific emigrant groups, larger effects on the percentage changes in Russia could have been expected. But the emigrant groups considered here accounted for only 0.8% of the total population in Russia in 2007. Taking healthy migrant effects into consideration, which may accompany the in-migration of all the other large and often fairly specific migrant groups in Russia, may amplify this small observed effect. Hence, the high CVD mortality measures reported by the WHO for Russia might even somewhat underestimate the true CVD mortality rate in the original Russian population.

On the other hand, the mortality rate in Russia may also have been affected by immigration from other eastern countries with high mortality rates, which may have cancelled out or even reversed the presented effect. However, since Russia has one of the highest reported CVD mortality rates in recent decades, if we assume the healthy migrant effect would hold for migrants from Eastern Europe and Asian countries, such a reversal is unlikely.

A recent review of environmental and societal influences on CVD risk factors found that the dramatic falls in mortality trends in some countries, as well as the typical differences in patterns between countries, are largely driven by risk factor changes on the population level, combined with effects of improved interventions and better curative care [32]. The examples of Russia and Germany illustrate these assumptions. However, our findings show that migration has a measurable effect on the decline of CVD mortality rates as well, especially in industrialized countries with non-negligible immigration of specific subgroups. Considering the migration process in Germany over the last 30 years, as well as the healthy migrant effect, immigration may have contributed to some extent to the halving of the CVD mortality rate. In other countries like the USA, with larger and increasing levels of immigration by migrants with low CVD mortality rates, for example migrants from Mexico, migration may even explain a larger proportion of the decline in mortality.

In Russia, risk factor changes at the population level were mirrored by the mortality trends. The reduction in the overall mortality rate in 1985 and afterwards in Russia was partly due to a nationwide anti-alcohol campaign which reduced external death rates first and CVD death rates thereafter [25,33,34]. The steep increase in all CVD-related deaths after 1992, also observed in the overall mortality rate, is not an artefact since this pattern was not observed in deaths caused by neoplasm, but rather it was mostly influenced by rising alcohol consumption after the anti-alcohol campaign ended [33]. Additionally, CVD-related deaths caused by stress due to the socioeconomic circumstances increased massively. Between 1995 and 1998 the economic prospects in Russia improved, resulting in lower stress levels in the population, followed once again by worsening living conditions. In summary, the CVD-related deaths in Russia played an important role following the collapse of the FSU due to emotional stress and increasing poverty. Recent CVD mortality trends in Russia seem to be more affected by trends in the economy. The influence of politics can also be seen in the birth rate, where political uncertainty led to high abortion rates in Russia in the 1990s [35,36].

A marginal part of the variation within the CVD time pattern in Russia may also have been caused by technical problems in ascertaining the causes of death due to conditions present during the early 1990s. However, the total mortality in this period, where technical problems were unlikely, showed a similar pattern.

There were some limitations in our data. Information on German repatriates who left Russia could not be obtained for the years before 1992 (we used estimates before 1992), and for Jews before 1990. Due to the small numbers involved here we do not think that this had a major impact on the results.

An east-west gradient of CVD mortality may in fact also be present within large countries like Russia, and German repatriates and Jewish emigrants may have originated mainly from areas with lower mortality rates. Since we only corrected nationwide reported mortality rates, our analysis did not consider a possible skewed distribution of German repatriates and Jewish emigrants in Russia.

Other limitations in our work were the assumption of constant SMRs for the migrants over time and the use of SMR estimates from former studies without considering the range of the confidence intervals in estimating the effects. But the SMRs of CVD were relatively stable during the observed calendar period. Therefore, using the constant overall SMR for the estimation procedure is acceptable.

When calculating the percentage changes in CVD trends, we used the weights of the subgroups in comparison to the total population for each, assuming that the gender ratio was balanced. However, in Russia for instance, it is known that the population in 2000 was comprised of about 7% more women than men [25]. Thus, although the calculated percentage changes may be somewhat biased due to the gender imbalance, we do not see this as a major source of error either.

## Conclusions

The east-west gap in CVD mortality rates within Europe has been increasing until recently. Trends in Russia and Germany were affected by the migration that occurred between both countries over recent decades; however, this effect was moderate.

On the one hand, knowledge about the health status and risk profiles of migrants is necessary for developing subgroup-specific public health strategies and to sensitize the health care community to the particular health problems of migrants. On the other hand, migration processes and their influences on aggregate measurements like mortality rates in health reporting should be considered when drawing conclusions regarding mortality rates in the total population.

The migration of large specific subgroups is an additional factor influencing national mortality dynamics. Interpreting mortality statistics while ignoring the bias caused by migration processes may lead to the misinterpretations of turning points in time trends as well as over- or underestimations of rates in the population of origin. These findings are important when using mortality rates as an indicator for evaluating nationwide public health programmes.

## Abbreviations

BVA: Bundesverwaltungsamt; CVD: Cardiovascular disease; FSU: Former Soviet Union; HFA-DB: European Health For All Database; SB: Statistisches Bundesamt; SMR: Standardized mortality ratio; WHO: World Health Organization

## Competing interests

The authors declare that they have no competing interests.

## Authors' contributions

AD obtained the data, performed the calculations, developed the figures and drafted the manuscript. VW provided the SMR estimates and knowledge based on his prior work and helped in drafting the manuscript. AP provided data, contributed his knowledge about the migrants from Russia and helped in interpreting the time trends. OR critically supported drafting of the manuscript and helped in elaborating details. HB conceived the study and contributed to the calculations and to drafting the manuscript. All authors read and approved the final manuscript.

## Pre-publication history

The pre-publication history for this paper can be accessed here:

http://www.biomedcentral.com/1471-2458/10/488/prepub
